# Elevated levels of TNF-α, IL-1β and IL-6 in the synovial tissue of patients with labral tear: a comparative study with hip osteoarthritis

**DOI:** 10.1186/s12891-020-03888-w

**Published:** 2021-01-06

**Authors:** Tomohisa Koyama, Kentaro Uchida, Kensuke Fukushima, Yoshihisa Ohashi, Katsufumi Uchiyama, Gen Inoue, Naonobu Takahira, Masashi Takaso

**Affiliations:** 1 Department of Orthopedic Surgery, Kitasato University School of Medicine, 1-15-1 Minami-ku, Kitasato, Sagamihara City, Kanagawa 252-0374 Japan; 2 Department of Rehabilitation, Kitasato University School of Allied Health Sciences, 1-15-1 Minami-ku, Kitasato, Sagamihara City, Kanagawa 252-0374 Japan

**Keywords:** Labral tear, Synovial tissue, TNF-α, IL-1β, IL-6

## Abstract

**Background:**

Labral tear can be the initiating factor in the onset of hip osteoarthritis (HOA). However, the physiopathology of labral tear is not fully understood. Our aim was to compare synovial tissue inflammatory cytokine levels between patients with labral tear and late-stage HOA.

**Methods:**

Synovial tissue from sites showing the greatest inflammation was harvested from 106 hips from 100 subjects during hip surgery. RNA was extracted, and levels of *TNFA*, *IL1B*, *IL6* and *COX2* mRNA were compared among all patients using real-time PCR. Additionally, we examined whether femoroacetabular impingement (FAI) was associated with elevated levels of inflammatory cytokines in patients with labral tear. To analyze the effects of TNF-α on inflammatory mediators in hip synovial tissue, synovial fibroblasts were extracted from hip synovial tissue of patients with labral tear and late-stage HOA (*n* = 5 each). Mononuclear cells were extracted from synovial tissue, cultured for 7 days, and stimulated with control or 10 ng/mL human recombinant TNF-α for 1 day. mRNA was extracted from stimulated cells and *IL1B*, *IL6*, and *COX2* levels were determined using real-time PCR.

**Results:**

*TNFA*, *IL1B,* and *COX2* expression in synovial tissue were significantly higher in patients with labral tear than late-stage HOA (*TNFA*, *p* <  0.001; *IL1B*, *p* <  0.001; *COX2*, *p* = 0.001). There were no differences in expression between patients with labral tear with and without FAI (*TNFA*, *p* = 0.546; *IL1B*, *p* = 0.559; *IL6*, *p* = 0.599; *COX2*, *p* = 0.124). Compared to vehicle control, TNF-α stimulation significantly elevated *IL1B*, *IL6,* and *COX2* expression in synovial fibroblasts collected from patients with labral tear and late-stage HOA (*IL1B*, *p* = 0.043 and *p* = 0.043; *IL6*, *p* = 0.043 and 0.043; *COX2*, *p* = 0.043 and *p* = 0.080, respectively).

**Conclusions:**

*TNFA*, *IL1B,* and *COX2* expression were elevated in the synovial tissue of patients with labral tear. Further investigations are needed to reveal the relationship between inflammatory cytokine levels and various aspects of labral tear pathology, including pain and the onset and progression of OA.

**Supplementary Information:**

The online version contains supplementary material available at 10.1186/s12891-020-03888-w.

## Background

The acetabular labrum is necessary for hip-joint stability, [1] and loss of structural integrity of the labrum leads to hip pain, subluxation, or instability [2, 3]. A labral tear (LT) can be preceded by femoroacetabular impingement (FAI), hip dysplasia, or hip osteoarthritis (HOA) [4]. LT can also lead to articular cartilage damage and the early stages of HOA [5]. However, the physiopathology of LTs is not fully understood.

Studies suggest that synovial inflammation is a key factor in the pain induced by LT, and in the initiation and progression of osteoarthritis (OA) induced by it [6–9]. Almost all reports published on the pathology of synovial inflammation have been conducted on the knee. However, a recent study reported that labral degeneration is positively correlated with synovial inflammation in patients with FAI [10]. Therefore, synovial inflammation may explain the occurrence of hip pain and OA progression in patients with LT. However, synovial inflammation in LT has not been fully characterized.

Tumor necrosis factor-α (TNF-α) plays a pivotal role in synovial inflammation and contributes to OA pathology [11]. It also induces other inflammatory mediators such interleukin (IL)-1β and cyclooxygenase-2 (COX-2) [12]. We previously reported that TNF-α levels were significantly elevated in patients who received arthroscopic surgery and showed HOA progression after surgery compared to those who did not show HOA progression [13]. However, because this study did not include a control group, further studies are needed to confirm whether TNF-α is elevated in patients with LT.

In this study, we aimed to compare synovial tissue (ST) expression of inflammatory cytokines between patients with LT and late-stage HOA. Specifically, we extracted ST from patients with LT who received arthroscopic surgery and those with late-stage HOA who received total hip arthroplasty (THA), and examined the mRNA levels of *TNFA, IL1B, IL-6,* and *COX2*.

## Methods

### Subjects

This study was approved by our Institutional Review Board (approval number: B19–259) and conducted based on the ethical standards of the 1964 Declaration of Helsinki and its later amendments. Written consent to participate was obtained from all participants. A total of 130 subjects who had ST samples extracted during an operation to treat HOA from October 2013 to March 2019 were enrolled. All subjects received non-steroidal anti-inflammatory drugs (NSAIDs) preoperatively. We excluded subjects with inflammatory diseases, including neoplastic disease and rheumatoid arthritis, and those with a history of hip surgery. Further, subjects were also excluded if examination of their samples failed due to laboratory error. Finally, we included 106 hips from 100 patients, with 32 hips with LT receiving hip arthroscopic surgery and 74 hips with late-stage OA receiving THA. HOA grade was assessed radiographically according to the Kellgren-Lawrence (K-L) classification [14]. Pain at rest and on activity was scored on a 0 (no pain) to 10 (worst pain imaginable) cm visual analog scale (VAS). In patients who received hip arthroscopy, we also determined the presence of FAI according to the proposed diagnostic criteria of the Japan Hip Society [15].

### Methods

ST samples were extracted based on the gross and arthroscopic appearance of sites showing redness during surgery. A single experienced clinician performed all synovial tissue extractions and radiological assessments throughout the study. ST samples were immediately transferred to liquid nitrogen before storage in a freezer at − 80 °C until use for RNA extraction. RNA was extracted from ST samples, and levels of *TNFA*, *IL1B*, *IL6,* and *COX2* mRNA were compared among all patients using real-time polymerase chain reaction (PCR). ST samples extracted from 10 patients (*n* = 5 each) were immediately digested using collagenase and used for cell culture.

### Real-time polymerase chain reaction (PCR)

ST samples and cultured synovial cells were subjected to a total RNA extraction process using TRIzol (Invitrogen, Carlsbad CA, USA) according to the manufacturer’s instructions. The resulting RNA formed the template for first-strand cDNA synthesis of *TNFA*, *IL1B*, *IL6,* and *COX2.* Briefly, Moloney murine leukemia virus reverse transcriptase (SuperScript III RT, Invitrogen) was used in 25-μL reaction mixtures comprising 2 μL cDNA, 0.2 μM specific primer pair, 12.5 μL N′,N′-dimethyl-N-[4-[(E)-(3-methyl-1,3-benzothiazol-2-ylidene)methyl]-1-phenylquinolin-1-ium-2-yl]-N-propylpropane-1,3-diamine dye reagent (SYBR Premix Ex Taq, Takara, Kyoto, Japan), and nuclease-free water. Primers were designed in Primer Blast (http:// www.ncbi.nlm.nih.gov/tools/primer-blast/) and synthesized by Hokkaido System Science Co., Ltd. (Sapporo, Japan). PCR primer pair sequences are shown in Table 1. Amplified sequences were investigated for specificity using melt curve analysis. Quantitative PCR conducted using a Real-Time PCR Detection System (CFX-96; Bio-Rad, CA, USA) was used to examine relative mRNA expression. PCR cycle parameters were as follows: 95 °C for 1 min, followed by 40 cycles at 95 °C for 5 s and 60 °C for 30 s. mRNA levels of test genes were normalized to those of glyceraldehyde-3-phosphate dehydrogenase (*GAPDH*). Correlations between gene expression and BMI and VAS were examined.

**Table 1:** Primer sequences

Gene	Direction	Primer sequence (5′–3′)	Product size (bp)
*TNFA*	Sense	CTTCTGCCTGCTGCACTTTG	118
	Antisense	GTCACTCGGGGTTCGAGAAG	
*IL1B*	Sense	GTACCTGTCCTGCGTGTTGA	153
	Antisense	GGGAACTGGGCAGACTCAAA	
*IL6*	Sense	GAGGAGACTTGCCTGGTGAA	199
	Antisense	TGGCATTTGTGGTTGGGTCA	
*COX2*	Sense	TGGCTGAGGGAACACAACAG	74
	Antisense	AACAACTGCTCATCACCCCA	
*GAPDH*	Sense	TGTTGCCATCAATGACCCCTT	202
	Antisense	CTCCACGACGTACTCAGCG	

### Synovial cell culture

To determine whether TNF-α is involved in regulating inflammatory cytokines (IL1β, IL-6) and COX-2, synovial fibroblasts were extracted from hip ST removed from patients with LT and late-stage HOA (*n* = 5 each). Mononuclear cells were extracted from ST samples by digestion with 20 mL of 0.1% type I collagenase, and were subsequently cultured in α-minimal essential media (ɑ-MEM) supplemented with 10% fetal bovine serum at 1 × 10^4^ cells/cm^2^ in six-well plates for 7 days. We confirmed that almost all cultured cells (> 90%) were positive for the synovial fibroblast marker, CD90, and negative for the pan hematopoietic marker, CD45, using flow cytometry (Additional file 1: Figure S1). After the 7 days in culture, passage 0 synovial cells were subjected to stimulation with control (ɑ-MEM) or 10 ng/mL human recombinant TNF-α for 1 day. To analyze the effects of TNF-α stimulation, mRNA was extracted from stimulated cells and *TFNA, IL1B, IL6*, and *COX2* levels were determined using real-time PCR.

### Statistical analysis

SPSS version 19.0 (SPSS, IL, Chicago, USA) was used for statistical analysis. Numerical results are shown as mean and standard error of the mean unless otherwise indicated. The Kolmogorov-Smirnov test was used to determine whether or not the data were normally distributed. Patients’ clinical characteristics were compared between the LT and late-stage HOA groups as categorical and continuous variables using the chi-squared test and unpaired t-test, respectively. Gene expression levels were compared between patients with LT and late-stage HOA using the Mann-Whitney U test. The relationship between gene expression levels and BMI and VAS was analyzed using Spearman’s correlation coefficient. For comparisons of stimulated synovial fibroblasts, *P* <  0.05 was used to indicate statistical significance.

## Results

### Clinical characteristics of patients with LT and late-stage HOA

Patients with LT were significantly younger than those with late-stage HOA (*p* <  0.001). VAS score on walking was significantly higher in patients with late-stage HOA than LT (*P* = 0.024). There was no significant difference in VAS score at rest (*P* = 0.636). The proportion of patients with each K-L grade differed significantly between HOA and LT groups (*P* <  0.001). All patients with LT had K-L Grade 1 or 2 and underwent arthroscopy, with 27 cases receiving suture anchors and the others receiving arthroscopic debridement and synovium resection. FAI was identified in 12 (37.5%) patients with LT. All patients with late-stage OA had K-L Grade 3 or 4 and underwent THA (Table 2).

**Table 2:** Patient characteristics

	Labral tear	Late-stage HOA	*P* value
Male/female	9/23	10/64	0.073
Age (years)	48.0 ± 9.8	64.4 ± 11.3	< 0.001*
BMI (kg/m^2^)	23.2 ± 0.5	23.7 ± 0.5	0.700
AS/THA	32/0	0/74	< 0.001*
VAS rest	3.4 ± 3.3	3.6 ± 3.0	0.636
VAS gait	4.8 ± 3.1	6.3 ± 2.5	0.024*
Kellgren-Lawrence classification (0/1/2/3/4)	0/18/14/0/0	0/0/0/10/64	< 0.001*

*HOA* hip osteoarthritis; *BMI* body mass index; *AS* arthroscopic surgery; *THA* total hip arthroplasty; *VAS* visual analogue scale

*P < 0.05

### Expression of inflammatory mediators in patients with LT

**Fig. 1:**
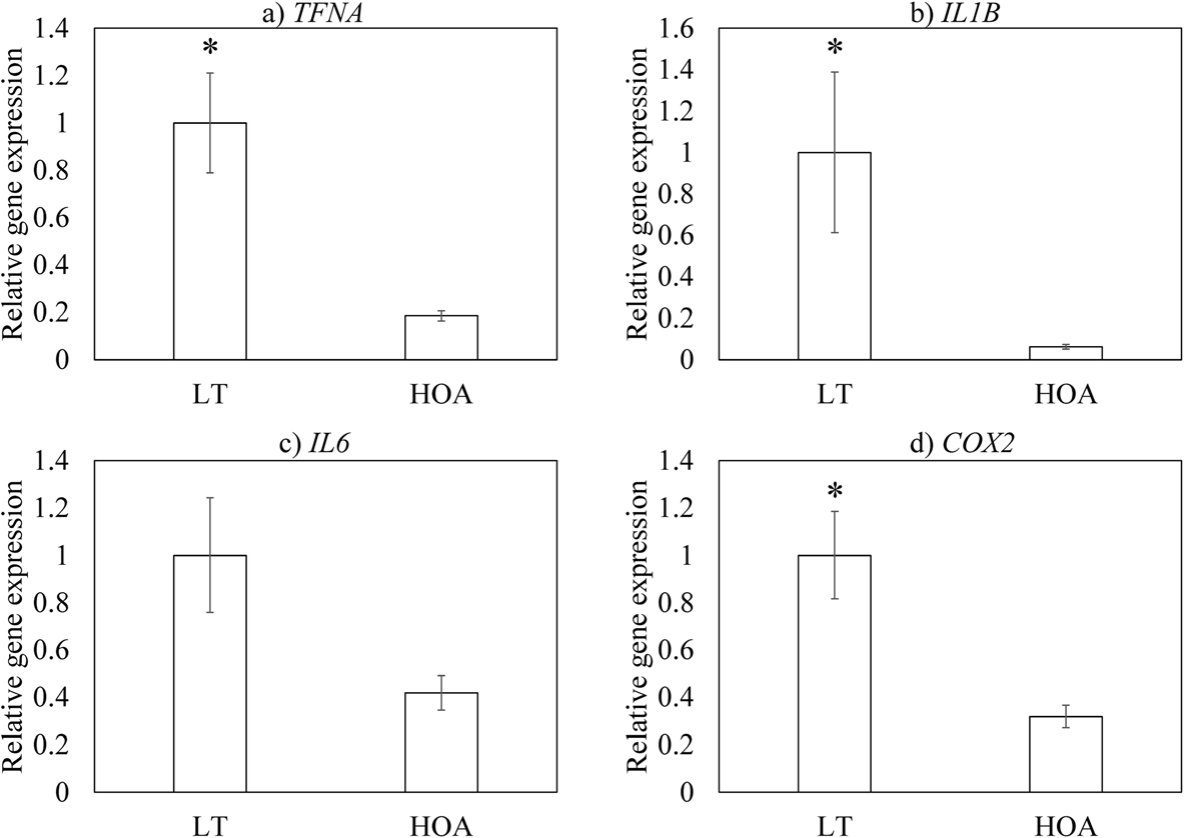
Expression of inflammatory mediators in synovial tissue from patients with LT and hip osteoarthritis (HOA). **a** *TNFA*, **b** *IL1B*, **c** *IL6*, and **d** *COX2*

*TNFA*, *IL1B,* and *COX2* mRNA levels in ST were significantly higher among patients with LT than those with late-stage HOA (*TNFA*, *p* <  0.001; *IL1B*, *p* < 0.001; *COX2*, *p* = 0.001; Fig. 1). In contrast, no significant differences were observed in *IL6* mRNA levels between patients with LT and late-stage HOA (*p* = 0.078).

### Expression of inflammatory mediators in patients with LT with and without FAI

**Fig. 2:**
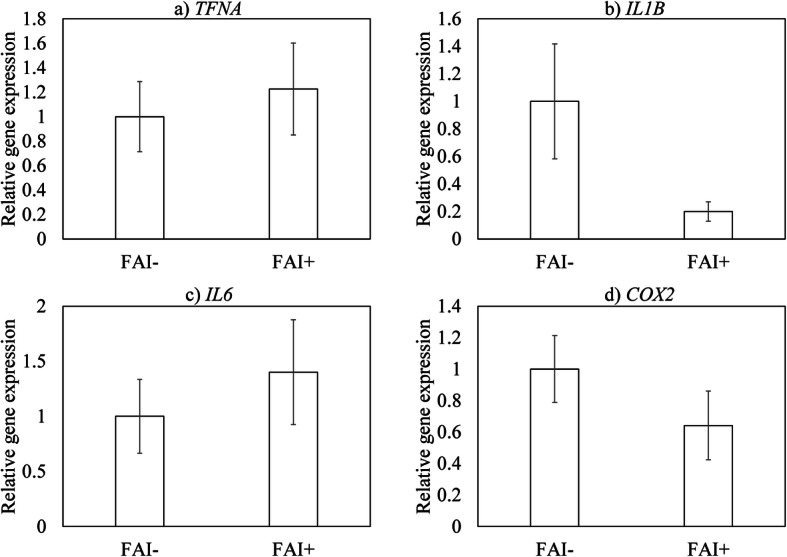
Expression of inflammatory mediators in synovial tissue from patients with LT with and without femoroacetabular impingement (FAI). **a** *TNFA*, **b** *IL1B*, **c** *IL6*, and **d** *COX2*

Previous studies have reported that inflammatory cytokine levels are elevated in the synovium of patients with FAI [13]. We therefore examined whether FAI was associated with elevated levels of *TNFA*, *IL1B*, *IL6* and *COX2*. We found no difference in the expression of these genes between patients with LT with and without FAI (*TNFA*, *p* = 0.546; *IL1B*, *p* = 0.559; *IL6*, *p* = 0.599; *COX2*, *p* = 0.124; Fig. 2).

### Effect of patients’ demographics on expression of inflammatory mediators among patients with LT

No correlation was found between *TNFA*, *IL1B*, *IL6* and *COX2* expression levels and VAS and BMI in either the LT or HOA group (Table 3). However, *IL6* expression was positively correlated with BMI in the LT group (ρ = 0.403, *P* = 0.022; Table 3).

**Table 3:** Correlation between in *TNFA*, *IL1B*, *IL6* and *COX2* expression levels and VAS and BMI in both labral tear and hip osteoarthritis groups

	VAS rest		VAS gait		BMI	
	LT	HOA	LT	HOA	LT	HOA
TNF-α	*P* = 0.818	*P* = 0.891	*P* = 0.863	*P* = 0.443	*P* = 0.673	*P* = 0.161
	*ρ* = − 0.042	*ρ* = 0.016	*ρ* = 0.032	*ρ* = − 0.091	*ρ* = 0.078	*ρ* = − 0.165
IL-1β	*P* = 0.526	*P* = 0.191	*P* = 0.640	*P* = 0.766	*P* = 0.434	*P* = 0.212
	*ρ* = −0.116	*ρ* = − 0.154	*ρ* = − 0.086	*ρ* = − 0.035	*ρ* = 0.143	*ρ* = − 0.147
IL-6	*P* = 0.913	*P* = 0.086	*P* = 0.583	*P* = 0.950	*P* = 0.022*	*P* = 0.383
	*ρ* = −0.020	*ρ* = − 0.201	*ρ* = 0.101	*ρ* = 0.007	*ρ* = 0.403	*ρ* = −0.103
COX-2	*P* = 0.305	*P* = 0.062	*P* = 0.966	*P* = 0.911	*P* = 0.358	*P* = 0.334
	*ρ* = 0.187	*ρ* = −0.218	*ρ* = 0.008	*ρ* = 0.013	*ρ* = 0.168	*ρ* = −0.114

*VAS* visual analogue scale; *BMI* body mass index; *LT* labral tear; *HOA* hip osteoarthritis

*P < 0.05

### Effect of BMI on pain among patients with LT

BMI was correlated with gait pain in the HOA group (*ρ* = 0.292, *P* = 0.012; Table 4) but not in the LT group (*ρ* = − 0.130, *P* = 0.479; Table 4). No correlation was observed between pain at rest and BMI in either the LT (*ρ* = − 0.050, *P* = 0.746; Table 4) or HOA group (*ρ* = 0.341, *P* = 0.112; Table 4).

**Table 4:** Correlation between VAS and BMI in labral tear and hip osteoarthritis groups

	VAS rest		VAS gait	
	LT	HOA	LT	HOA
BMI	*P* = 0.746	*P* = 0.112	*P* = 0.479	*P* = 0.012*
	*ρ* = − 0.050	*ρ* = 0.341	*ρ* = − 0.130	*ρ* = 0.292

*VAS* visual analogue scale; *BMI* body mass index; *LT* labral tear; *HOA* hip osteoarthritis

*P < 0.05

### Effect of TNF-α on IL1B, IL6 and COX2 expression

**Fig. 3:**
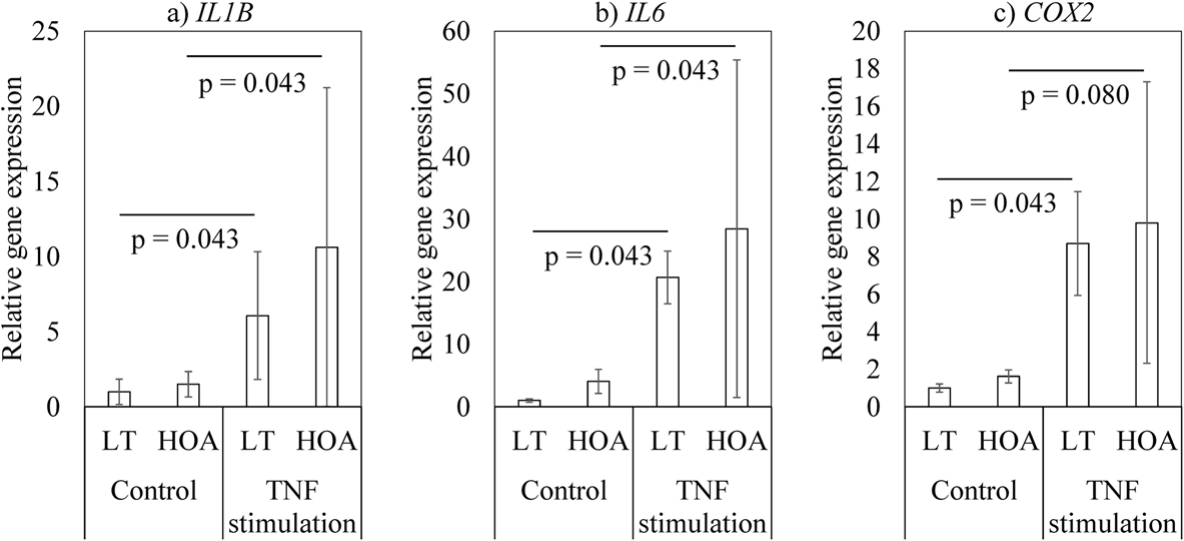
Effect of TNF-α stimulation on *IL1B*, *IL6*, and *COX2* expression in synovial cells derived from patients with LT and hip osteoarthritis. **a** *IL1B*, **b** *IL6*, and **c** *COX2*

Given that we found differences in the expression of inflammatory mediators between patients with LT and late-stage HOA, we next evaluated whether synovial cells were functionally different between these groups of patients. Age and the proportion of patients with each K-L grade differed significantly between HOA and LT patients (age, *P* < 0.001; K-L grade, *P* < 0.001; Table 5). Compared to vehicle control, TNF-α stimulation significantly increased inflammatory cytokine (*IL1B*, *IL6*) and *COX2* expression in synovial fibroblasts collected from patients with LT and late-stage HOA (*IL1B*, *p* = 0.043 and *p* = 0.043; *IL6*, *p* = 0.043 and 0.043; *COX2*, *p* = 0.043 and *p* = 0.080, respectively; Fig. 3). However, no differences in mRNA levels were observed between LT- and HOA-derived cell cultures following TNF-α stimulation.

**Table 5:** Characteristics of patients from whom synovial fibroblasts were harvested for synovial culture

	Labral tear	Late-stage HOA	P value
Male/female	3/2	0/5	0.050
Age (years)	45.8 ± 2.7	63.8 ± 1.6	0.009*
BMI (kg/m^2^)	23.9 ± 1.4	21.5 ± 1.5	0.172
AS/THA	5/0	0/5	< 0.001*
VAS rest	1.3 ± 0.5	1.6 ± 0.5	0.832
VAS gait	4.5 ± 0.9	7.0 ± 1.1	0.172
Kellgren-Lawrence classification (0/1/2/3/4)	0/5/0/0/0	0/0/0/0/5	0.003*

*HOA* hip osteoarthritis; *BMI* body mass index; *AS* arthroscopic surgery; *THA* total hip arthroplasty; *VAS* visual analogue scale

*P < 0.05

## Discussion

Despite accumulating evidence indicating that LTs are associated with OA progression and pain, the mechanism that drives the underlying pathology remains largely unknown [16]. TNF-α and IL-1β are major proinflammatory cytokines involved in synovial inflammation and contribute to cartilage catabolism and pain [17–19]. Here, we found that patients with LT have higher expression levels of *TNFA*, *IL1B*, and *IL6* than patients with late-stage HOA. In knee OA, synovial TNF-α and IL-1β expression are elevated in early compared to late-stage OA and correlate with early OA pathology [20]. In the present study, elevated levels of *TNFA* and *IL1B* were observed even in the pre-arthritic condition in the hip joint. TNF-α is correlated with pain in patients who have undergone arthroscopy [13]. However, LT patients had higher levels of inflammatory cytokines but a lower pain score compared to the HOA group. Further, that gait VAS correlated with BMI in the HOA group but not the LT group suggests that different factors, such as mechanical overloading, may contribute to OA pain in these patients. Further investigations are needed to determine how an increase in inflammatory mediators is associated with LT pathology.

FAI syndrome is an important source of pre-arthritic hip pain and secondary HOA [21]. LTs are commonly found in those with FAI [22]. Indeed, Trisolino et al. reported the presence of LTs in 86% of patients with FAI [10]. In the present study, 37.5% of patients with LT had FAI. However, the expression of inflammatory mediators did not differ between patients with and without radiographic signs of FAI. A recent study showed that labral histological degeneration is associated with an increase in CD68-positive cells in the synovium and leads to worsening of the post-operative outcome following arthroscopic surgery among patients with FAI [10]. Taken together, these previous and present results suggest that elevated synovial inflammatory cytokine levels may reflect the pathology of LT but not FAI.

The outcomes of ST inflammation are quantifiably distinct between early and late-stage HOA. Studies have additionally examined whether synovial fibroblasts from patients with early and late-stage OA are functionally distinct [20]. In this study, we stimulated cultured synovial fibroblasts with TNF-α and measured inflammatory cytokine levels. We found that stimulation with TNF-α significantly increased inflammatory cytokine (*TFNA*, *IL1B*, *IL6*) and *COX2* expression in synovial fibroblasts from patients with LT and late-stage HOA compared to unstimulated control cells. We saw no functional difference between synovial fibroblasts harvested from patients with early and late HOA. An increase in monocyte recruitment has been observed in early-stage knee OA [20]. A histological study showed that there is an increase in CD68-positive cells and vascularization in patients with FAI with labral degeneration [10]. Synovial macrophages have higher TNF-α and IL-1β expression than synovial fibroblasts in knee OA [23]. The higher expression of proinflammatory mediators in patients with LT may indicate differences in the ST microenvironment.

Various factors including age, gender and BMI have effects on cytokine expression [24–27]. Consistent with this, we observed a correlation between BMI and *IL6* expression in LT patients. Therefore, differences in patient demographics between the LT and HOA group may have skewed our results. Further investigation adjusting for these confounding factors is needed.

Several limitations of this study warrant mention. First, major limitation of this study is that the only measure used was PCR. Protein profiling studies such as western blotting and immunohistochemical analysis are needed to validate our gene expression profile results. Second, inclusion of a healthy control population is needed as an ideal control group. Third, it is unclear whether the change in cytokine expression is the cause or the result of the pathological condition observed by arthroscopy. Fourth, all patients received NSAIDs preoperatively. The effect of preoperative NSAIDs on gene expression is unclear. Fifth, changes in gene expression observed in this study may be related to the region sampled rather than pathology. Finally, it is unclear which cell types expressed the elevated levels of cytokines. Further research into these questions is warranted.

## Conclusion

*TNFA, IL1B* and *COX2* were elevated in the ST of patients with LT. LT was associated with increased synovial inflammatory cytokine levels. Further investigations are needed to reveal the relationship between inflammatory cytokine levels and various aspects of LT pathology, including pain and the onset and progression of OA.

## Supplementary Information

**Additional file 1 Figure S1**. Flow cytometric analysis of synovial cells obtained from labral tear (LT) and hip osteoarthritis (HOA) patients. (A and B) Dot‐plot analysis of CD45+CD90- and CD45- CD90+ cells among LT-derived (A) and HOA-derived (B) synovial cells. X‐axis, CD90; y‐axis, CD45.

## Data Availability

The datasets supporting the conclusions of this article are included within the article. The raw data can be requested from the corresponding author.

## Abbreviations

LT: Labral tear
FAI: Femoroacetabular impingement
HOA: Hip osteoarthritis
OA: Osteoarthritis
TNF-α: Tumor necrosis factor-α
IL: Interleukin
COX-2: Cyclooxygenase-2
ST: Synovial tissue
THA: Total hip arthroplasty
K-L classification: Kellgren-Lawrence classification
PCR: Polymerase chain reaction
GAPDH: Glyceraldehyde-3-phosphate dehydrogenase
VAS: Visual analog scale

## References

[1] Grant AD, Sala DA, Davidovitch RI (2012). The labrum: structure, function, and injury with femoro-acetabular impingement. J Child Orthop.

[2] Eijer H, Hogervorst T (2017). Femoroacetabular impingement causes osteoarthritis of the hip by migration and micro-instability of the femoral head. Med Hypotheses.

[3] Pitto RP, Klaue K, Ganz R, Ceppatelli S (1995). Acetabular rim pathology secondary to congenital hip dysplasia in the adult. A radiographic study. Chir Organi Mov.

[4] Parmar R, Parvizi J (2010). The multifaceted etiology of acetabular labral tears. Surg Technol Int.

[5] Klennert BJ, Ellis BJ, Maak TG, Kapron AL, Weiss JA (2017). The mechanics of focal chondral defects in the hip. J Biomech.

[6] Loeser RF (2006). Molecular mechanisms of cartilage destruction: mechanics, inflammatory mediators, and aging collide. Arthritis Rheum.

[7] Samuels J, Krasnokutsky S, Abramson SB (2008). Osteoarthritis: a tale of three tissues. Bull NYU Hosp Jt Dis.

[8] Sellam J, Berenbaum F (2010). The role of synovitis in pathophysiology and clinical symptoms of osteoarthritis. Nat Rev Rheumatol.

[9] Sokolove J, Lepus CM (2013). Role of inflammation in the pathogenesis of osteoarthritis: latest findings and interpretations. Ther Adv Musculoskelet Dis.

[10] Trisolino G, Favero M, Dallari D, Tassinari E, Traina F, Otero M, Goldring SR, Goldring MB, Carubbi C, Ramonda R, Stilli S, Grigolo B, Olivotto E (2020). Labral calcification plays a key role in hip pain and symptoms in femoroacetabular impingement. J Orthop Surg Res.

[11] Smith MD, Triantafillou S, Parker A, Youssef PP, Coleman M (1997). Synovial membrane inflammation and cytokine production in patients with early osteoarthritis. J Rheumatol.

[12] Wojdasiewicz P, Poniatowski LA, Szukiewicz D. The role of inflammatory and anti-inflammatory cytokines in the pathogenesis of osteoarthritis. Mediat Inflamm. 2014;561459. 10.1155/2014/561459. 10.1155/2014/561459 PMC4021678 24876674

[13] Fukushima K, Inoue G, Uchida K, Fujimaki H, Miyagi M, Nagura N, Uchiyama K, Takahira N, Takaso M (2018). Relationship between synovial inflammatory cytokines and progression of osteoarthritis after hip arthroscopy: Experimental assessment. J Orthop Surg (Hong Kong ).

[14] KELLGREN JH, LAWRENCE JS (1957). Radiological assessment of osteo-arthrosis. Ann Rheum Dis.

[15] Mimura T, Mori K, Itakura S, Furuya Y, Kawasaki T, Imai S (2017). Prevalence of pincer, cam, and combined deformities in Japanese hip joints evaluated with the Japanese hip society diagnostic guideline for femoroacetabular impingement: a CT-based study. J Orthop Sci.

[16] Schmerl M, Pollard H, Hoskins W (2005). Labral injuries of the hip: a review of diagnosis and management. J Manip Physiol Ther.

[17] Goldring MB, Otero M (2011). Inflammation in osteoarthritis. Curr Opin Rheumatol.

[18] Hosny S, Strambi F, Sofat N, Field R. A systematic review investigating the presence of inflammatory Synovitis in hip and knee joint replacement surgery. Arthritis. 2015;729410. 10.1155/2015/729410. 10.1155/2015/729410 PMC4628772 26557388

[19] Scanzello CR, Goldring SR (2012). The role of synovitis in osteoarthritis pathogenesis. Bone..

[20] Benito MJ, Veale DJ, FitzGerald O, van den Berg WB, Bresnihan B (2005). Synovial tissue inflammation in early and late osteoarthritis. Ann Rheum Dis.

[21] Zhang C, Li L, Forster BB, Kopec JA, Ratzlaff C, Halai L, Cibere J, Esdaile JM (2015). Femoroacetabular impingement and osteoarthritis of the hip. Can Fam Physician.

[22] Kautzner J, Havlas V, Trc T (2016). Femoroacetabular impingement treatment options. Cas Lek Cesk.

[23] Takano S, Uchida K, Inoue G, Miyagi M, Aikawa J, Iwase D, Iwabuchi K, Matsumoto T, Satoh M, Mukai M, Minatani A, Takaso M (2017). Nerve growth factor regulation and production by macrophages in osteoarthritic synovium. Clin Exp Immunol.

[24] Michaud M, Balardy L, Moulis G, Gaudin C, Peyrot C, Vellas B, Cesari M, Nourhashem F (2013). Proinflammatory cytokines, aging, and age-related diseases. Review J Am Med Dir Assoc.

[25] Loukov D, Karampatos S, Maly MR, Bowdish DME (2018). Monocyte activation is elevated in women with knee-osteoarthritis and associated with inflammation, BMI and pain. Osteoarthritis Cartilage.

[26] Xianpeng G, Ritter SY, Tsang K, Shi R, Takei K, Aliprantis AO (2016). Sex-Specific Protection of Osteoarthritis by Deleting Cartilage Acid Protein. PLoS One.

[27] Xue XT, Zhang T, Cui SJ, He DQ, Wang XD, Yang RL, Liu DW, Liu Y, Gan YH, Kou XX, Zhou YH (2018). Sexual dimorphism of estrogen-sensitized synoviocytes contributes to gender difference in temporomandibular joint osteoarthritis. Oral Dis.

