# Supplementary material for: Elevated levels of TNF-α, IL-1β and IL-6 in the synovial tissue of patients with labral tear: a comparative study with hip osteoarthritis
Source: BMC Musculoskelet Disord. 2021 Jan 6;22:33. doi: 10.1186/s12891-020-03888-w (PMC7788943; doi:10.1186/s12891-020-03888-w)
Supplement: Supplementary file 1 — Additional file 1 Figure S1. Flow cytometric analysis of synovial cells obtained from labral tear (LT) and hip osteoarthritis (HOA) patients. (A and B) Dot‐plot analysis of CD45+CD90- and CD45- CD90+ cells among LT-derived (A) and HOA-derived (B) synovial cells. X‐axis, CD90; y‐axis, CD45. [file 12891_2020_3888_MOESM1_ESM.docx]

Figure S1. Flow cytometric analysis of synovial cells obtained from labral tear (LT) and hip osteoarthritis (HOA) patients


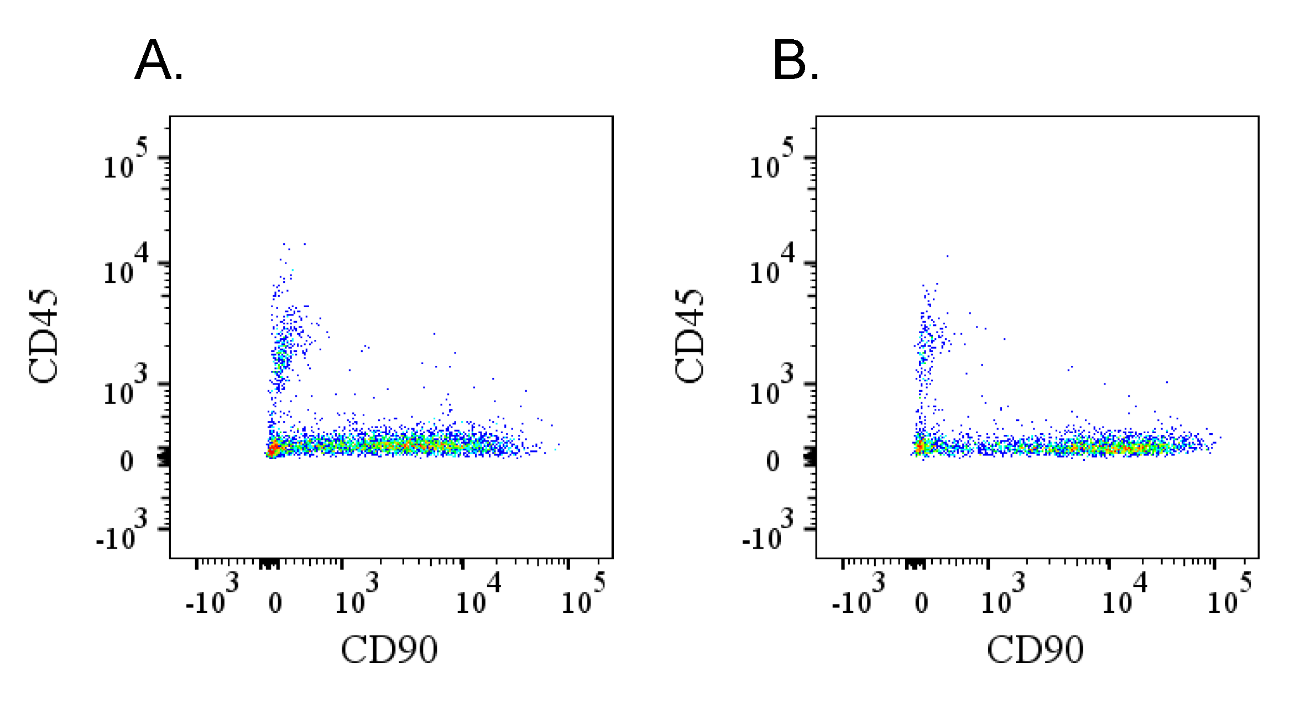


(A and B) Dot‐plot analysis of CD45+CD90- and CD45- CD90+ cells among LT-derived (A) and HOA-derived (B) synovial cells. X‐axis, CD90; y‐axis, CD45.
